# Discovery of Single Nucleotide Polymorphisms in Complex Genomes Using SGSautoSNP

**DOI:** 10.3390/biology1020370

**Published:** 2012-08-27

**Authors:** Michał T. Lorenc, Satomi Hayashi, Jiri Stiller, Hong Lee, Sahana Manoli, Pradeep Ruperao, Paul Visendi, Paul J. Berkman, Kaitao Lai, Jacqueline Batley, David Edwards

**Affiliations:** 1 Australian Centre for Plant Functional Genomics, School of Agriculture and Food Science, University of Queensland, Brisbane, QLD 4072, Australia; Email: m.lorenc@uq.edu.au (M.T.L.); leehongching@gmail.com (H.L.); m.sahana@uq.edu.au (S.M.); p.ruperao@uq.edu.au (P.R.); paul.muhindira@uqconnect.edu.au (P.V.); k.lai1@uq.edu.au (K.L.); 2 Centre for Integrative Legume Research, School of Agriculture and Food Science, University of Queensland, Brisbane, QLD 4072, Australia; Email: s.hayashi@uq.edu.au (S.H.); j.batley@uq.edu.au (J.B.); 3 CSIRO Plant Industry, Brisbane, QLD 4072, Australia; Email: Jiri.Stiller@csiro.au (J.S.); Paul.Berkman@csiro.au (P.J.B.); 4 Centre of Excellence in Genomics (CEG), International Crops Research Institute for the Semi-Arid Tropics (ICRISAT), Patancheru 502324, Andhra Pradesh, India

**Keywords:** single nucleotide polymorphisms, wheat, autoSNP, genome diversity, genotyping by sequencing, haplotype

## Abstract

Single nucleotide polymorphisms (SNPs) are becoming the dominant form of molecular marker for genetic and genomic analysis. The advances in second generation DNA sequencing provide opportunities to identify very large numbers of SNPs in a range of species. However, SNP identification remains a challenge for large and polyploid genomes due to their size and complexity. We have developed a pipeline for the robust identification of SNPs in large and complex genomes using Illumina second generation DNA sequence data and demonstrated this by the discovery of SNPs in the hexaploid wheat genome. We have developed a SNP discovery pipeline called SGSautoSNP (Second-Generation Sequencing AutoSNP) and applied this to discover more than 800,000 SNPs between four hexaploid wheat cultivars across chromosomes 7A, 7B and 7D. All SNPs are presented for download and viewing within a public GBrowse database. Validation suggests an accuracy of greater than 93% of SNPs represent polymorphisms between wheat cultivars and hence are valuable for detailed diversity analysis, marker assisted selection and genotyping by sequencing. The pipeline produces output in GFF3, VCF, Flapjack or Illumina Infinium design format for further genotyping diverse populations. As well as providing an unprecedented resource for wheat diversity analysis, the method establishes a foundation for high resolution SNP discovery in other large and complex genomes.

## 1. Introduction

Molecular genetic markers describe genetic variations and provide a link between observed phenotypes and the underlying genotype [1,2,3]. The development of high-throughput methods for the detection of single nucleotide polymorphisms (SNPs) has led to a revolution in their use as molecular markers [4,5,6,7]. SNPs may be considered the ultimate genetic marker as they represent the finest resolution of a DNA sequence, are generally abundant in populations and have a low mutation rate [8]. The principal challenge in SNP discovery remains the discrimination between true genetic polymorphisms and the often more abundant sequence or mapping errors. SNP discovery is further confounded in polyploid species where multiple related genomes are present within each nucleus. The identification of sequence errors can be based on three methods: sequence quality score, redundancy of the polymorphism in a sequence alignment and presence of multiple haplotypes at a locus [9,10]. SNP redundancy provides an effective means for estimating confidence in the validity of SNPs independently of sequence quality scores and has been demonstrated to be an accurate method for SNP discovery in a range of species [11,12,13].

Many plant genomes are large and complex. The bread wheat (*Triticum aestivum*) genome is 17 Gbp in size, around 6 times larger than the human genome [14], consists of 75%–90% repeats [15,16] and is hexaploid, containing of the A, B and D genomes, each with 7 homoeologous chromosomes. The presence of multiple genomes, large size and abundant repeats make any genetic and genomic analysis a challenge [17]. The recent shotgun sequence assembly of isolated chromosome arms for the group 7 chromosomes provides a reference for SNP discovery across these chromosomes [18,19,20]. When combined with the recently produced whole genome shotgun sequence data for several wheat cultivars [21], there is a potential to identify large numbers of cultivar specific SNPs. 

The rapidly expanding genome datasets, driven by advances in second generation DNA sequencing, present a challenge for their management and application [22]. The majority of SNP discovery software is designed for human or simple bacterial genomes, and they are not well adapted for polyploid plant genomes, especially crop genomes which are often highly homozygous [11,23,24,25]. Because of this, we have established a novel method for SNP discovery from complex genomes called SGSautoSNP, extended from original concepts developed in autoSNP, SNPServer and autoSNPdb [11,12,26]. Rather that attempting to identify all possible SNPs across a genome, SGSautoSNP aims to identify as many highly confident SNPs as possible with the acknowledgement that not all biologically present SNPs will be identified. Here, we present the application of SGSautoSNP to wheat chromosomes 7A, 7B and 7D to identify more than 800,000 SNPs with an accuracy of greater than 93%, and present these polymorphisms within a GBrowse genome viewer at the wheatgenome.info web site [27].

## 2. Results and Discussion

We have developed a novel pipeline for the discovery of SNP polymorphisms in complex genomes. The SGSautoSNP (Second-Generation Sequencing AutoSNP) pipeline calls single nucleotide polymorphisms (SNPs) between different individuals using Illumina paired read data aligned to a reference. SGSautoSNP uses BAM (Binary Alignment/Map) format in order to save memory and space. These SNPs can be viewed using a broad range of visualization tools using GFF3, VCF and Flapjack output files. There is often a requirement to generate a consensus sequence based on the reads mapped to the reference and so SGSautoSNP can generate a consensus sequence as well as marker design files Illumina GoldenGate of Infinium assay designs.

There are many SNP discovery tools available. The main difference between our approach and most other SNP callers such as ACCUSA [28], AGSNP [29], NGS-SNP [30] and Atlas-SNP2 [31] is that the SGSautoSNP method does not consider the reference for SNP discovery. Instead, the reference is used to assemble the reads, and SNPs are then called between these assembled reads. Another difference is that other SNP callers are designed for SNP discovery from heterozygous populations. However, crop plants are frequently homozygous. In SGSautoSNP, mismapped reads produce a heterozygous genotype call at a locus, allowing their distinction from true homozygous SNPs. The SGSautoSNP method does not consider read quality score because these scores are not very reliable, with erroneous nucleotide calls often having high quality scores. 

We demonstrate the potential of SGSautoSNP by searching for SNPs between four wheat cultivars using whole genome shotgun paired read Illumina sequence data. The reference consisted of bread wheat chromosome arm shotgun assemblies representing chromosomes 7A, 7B and 7D, as well as 4AL [32]. All three of the group 7 chromosome homoelogs were included in the mapping reference to ensure reads mapped to their correct homoeologous location. In the absence of one of the homoeologs, cultivar specific reads from the missing homoeolog would likely map to one of the other homoeologous genomes, confounding SNP discovery. An assembly from chromosome arm 4AL was included as this arm contains a reciprocal translocation with 7BS [18]. If 4AL was absent from the reference, 4AL specific reads which correspond to the 7BS translocation may map to the orthologous region on 7AS or 7BS, again confounding SNP discovery. 

Cultivar specific reads were mapped to the reference sequences using SOAP [33]. The −r 0 option was applied which removes reads where they match multiple positions equally well. This option aims to increase SNP calling accuracy by ignoring read pairs that cannot be accurately positioned on the reference. Similarly, only reads that mapped as a pair were used for SNP discovery. Due to the short length of the reads, one read could match at many positions, but two reads separated by a gap of defined insert size provides a greater confidence of specific and accurate read mapping. The calling of SNPs between reads aligned to a reference while ignoring the reference allele allows this pipeline to be applied to accurately call SNPs between individuals using a reference from a divergent species. While this pipeline does not attempt to call all biological SNPs, the very large numbers of SNPs identified are valuable for genetic studies and the association of traits with candidate agronomic genes.

Between 661,600,411 and 899,700,085 genome sequence read pairs where generated for each of the four wheat cultivars (Table 1). Of these, between 4.70% and 3.10% mapped to the group 7/4AL reference as read pairs. As the reference is estimated to cover 14% of the complete genome, the number of mapped reads is less than predicted. This is likely due to many read pairs mapping to multiple locations in this highly repetitive genome and subsequently being ignored due to the SOAP –r 0 option.

SGSautoSNP identified a total of 881,289 SNPs across the group 7 chromosomes. These consisted of 68% transitions and 32% transversions. This bias in transition/transversion ratio is commonly observed in SNP discovery and reflects the high degree of methlyl C to U mutation in genomes [34]. It may be expected that the bread wheat genome is highly methylated due to the two rounds of polyploidy and high repeat content. The observed transition/transversion bias provides a level of confidence in SNP prediction accuracy since erroneously called SNPs caused by sequence read errors or mismapping would be unlikely to display such a bias. 

Validating individual SNPs in a hexaploid species is a challenge as the amplification of loci requires the design of homoeolog specific PCR primers. Of 40 SNPs selected for validation, 12 did not produce clean PCR amplification products or Sanger sequence. This reflects inefficiency in validation rather than SNP calling errors and so these SNPs were ignored. Of the 28 SNPs that did produce clean Sanger sequence data, 26 (93%) produced the expected genotype. One SNPs was homozygous across cultivars and not a true SNP, while one appeared to be heterozygous, suggesting a SNP between the homoeologous genomes rather than between cultivars.

All predicted SNPs have been included in a public wheat genome GBrowse database hosted at the wheatgenome.info web site [27]. This resource represents a substantial expansion over other recent wheat SNP discovery projects and provides a much greater density of SNPs than recently described methods for wheat. Allen *et al.* [35] recently identified 14,078 putative SNPs in 6,255 distinct reference sequences with Illumina GAIIx data from wheat lines Avalon, Cadenza, Rialto, Savannah and Recital. The validation rate from a subset of 1,659 was 67%. In a separate project, Lai *et al.* [36] identified a total of 38,928 candidate SNPs from bread wheat Roche 454 transcriptome data with an accuracy of 78% and presented these in an online database [37]. A pipeline package called AGSNP has also been applied to identify SNPs between two accessions of one of the diploid progenitors of bread wheat, *Aegilops tauschii*. Roche 454 sequencing of *A. tauschii* accession AL8/78 was combined with Applied Biosystems SOLiD sequencing of genomic DNA and cDNA from *A. tauschii* accession AS75 using AGSNP to identify a total of 497,118 candidate *A. tauschii* SNPs [29]. Currently, genome wide identification of hexaploid bread wheat SNPs using our pipeline is limited by the lack of publically available chromosome sequences. However, the identification of 881,289 SNPs across the group 7 chromosomes suggests that genome wide discovery would identify a total of more than 6 million SNPs.

The SNPs identified using SGSautoSNP can be used for genotyping by sequencing by low coverage skim sequencing of segregating wheat populations and calling genotypes where the low coverage sequence data aligns to a predicted polymorphic position. The successful application of the SGSautoSNP method to hexaploid wheat demonstrates that this approach should work for SNP discovery in other large and complex genomes.

**Table 1:** Summary of wheat cultivar data and mapping.

Wheat variety	Data generated	Data mapped to reference	% read pairs mapped
Drysdale	168 Gbp	8.65 Gbp	5.14%
Excalibur	146 Gbp	5.36 Gbp	3.66%
Gladius	180 Gbp	8.47 Gbp	4.70%
RAC875	132 Gbp	4.1 Gbp	3.10%

**Table 2:** Summary of Single nucleotide polymorphism (SNP) validation.

SNP Primer Name	Forward Primer	Reverse Primer	SNP score	Validation
UQ7A27	TAACATAAGCAAAGTTCTATTA	TTTGGAACACAATCGGAACTT	6	Failed
UQ7A1397	TCTATTGGATTCTTTCCGAT	TCACCCTGTGGAATGAAAGA	5	Failed
UQ7A5622	TTAGCCAAAATGGACCCAAA	CCTCTTTATTCAATCTGGAAACG	2	True SNP
UQ7A129835	TTCTTACTGTGGCTGCATCA	GCCATCCTAAACGACCTTCA	5	True SNP
UQ7A9400	GCCCATATGCAGTTCATGGT	AGAGCCAAACCTTCCCTGAT	2	Failed
UQ7A7915	CATGCCAACCCAAGTAGACC	GAAGCGTGAAAATTTCGTGA	6	True SNP
UQ7A6107	TGGTGTTTACGCTGAAGTTACC	CTGGCCTGGGCACTACATA	6	True SNP
UQ7A2603	GTCACCAACCAGCTCGAAAT	TTGTAGCTTTGCCTCTGTGAA	2	Failed
UQ7A3491	AGTCGCCGGCAGTAAAAATA	CCGAAGAAAATGTGGTGGAG	4	True SNP
UQ7A4532	TTTCCTCTAGATCTGTGCAAAATG	CATCCAGGACTGCATAAGCTC	6	True SNP
UQ7A100138	TCCCTGGTCCACGAGTTATT	AAATGGTTTGAGCCTTGTGC	7	Failed
UQ7A136305	CATCATCTTTGAAAAATCCTAGCC	TGTTCTGCAAGCTTCGTCTG	5	True SNP
UQ7A155877	AAGCTGTTGTGCCAGTGTTG	GAGCTAGCGTCGCTGACATA	4	True SNP
UQ7A180868	GACCGTCATCGAATGTAGCA	TCGTCCACCCAGACCTTATC	3	True SNP
UQ7A287189	GGCGATCATCACTTAAGAAACC	CAGTAATGAGGTTTCTGCTTGG	2	Failed
UQ7A322716	TCTGTTCGCAAACCAACG	GTGCGTTATCAGGGGAACAT	11	True SNP
UQ7A57227	ATGGGTGAAGGGAATACAGC	TGCATGCACATACAACCAAA	5	True SNP
UQ7A87191	TCAGTTCGGTAAGGATGAAGA	GAAGCAGTATGCATCTAAACTTTG	6	Heterozygous
UQ7B21	GCAGGGTTAATTTCTAGCAAGC	GCCTTTTATCCAAAGCCATC	8	Failed
UQ7B484	CTCAACCTCCCAAGCATGA	GCTATCCAGCTACCCTGTGC	11	Failed
UQ7B3940	GCCAGAGGCACTAGCATCAC	GGTAATTGTGGAGCAAGCAA	6	True SNP
UQ7B4960	GCATGGCATTTCAAGATCAG	GGAGGAGGACAAAGCCAGAT	5	True SNP
UQ7B5991	CCAAGCCACCACCCTTTAT	TAATCCCCGTCATCTCGAAG	4	True SNP
UQ7B120997	CTCCTCAGATGACCAATTTGC	CACCAAAATATGCTGTACAATTCTATG	7	Failed
UQ7B256895	GCAGCAGAGGTAGGCACTTC	GAAATGCTTCGAGTGTGGTG	11	True SNP
UQ7B64318	GGGTCCAGACTTCCACGTTA	CCCACATTAATTTGTACGACCTC	6	Failed
UQ7B97303	TGATTCGAGCCCATATAGGAA	AGCCATGCGGAAATATTGAG	8	True SNP
UQ7D283	TGAGTAAGACAACAATCAGAGCA	CAATGCGAGCAAAAAGATCA	5	True SNP
UQ7D429	TGTGCTGACGTGGCATCTAT	GCATGTGGAAAACGAGTGTG	3	True SNP
UQ7D689	CATCTGGCCTCAACATCAAA	TGTTGGTAGTGAGGCACTTCTT	9	Failed
UQ7D948	GGCGATACTCGATGAAAGAAA	TTGGAAACTACAATTGCACAAC	9	True SNP
UQ7D1189	GCGTGGAGTAGAGGGACAAG	TCCAAAAAGCAAAACAAATGC	4	True SNP
UQ7D1491	AGCGCAAGGAGGAGGTTAGT	GAGCCAAGTCCTTGTCAATTT	7	True SNP
UQ7D1846	AATGTGTTCCATCCAAGACG	GCCAAGGTCGACATGTGATA	10	True SNP
UQ7D2314	AAACAAGTCTGTGTTGCGTCA	TGCAGATACATGGCTCCAGA	2	Monomorphic
UQ7D20375	CTGCCACCAAACGGATTAAC	AATGCATTGGCAGTCACAAG	6	True SNP
UQ7D27168	TAATGCTATGCCGTGTCAGC	GCCACCTATTATTGAAGGCATC	2	True SNP
UQ7D38754	GAGCGAGCAATGCTAGTGTG	GAACCCATTTGATAACCGTGA	3	Failed
UQ7D59683	CGTCCACATTGTTGCAAATC	TTGACCCTGAAGGAAGGATG	6	True SNP
UQ7D68910	TTGCTTTATGCCACTGGAGA	TAGGCCGTGAAACATCAACA	3	True SNP

## 3. Experimental Section

### 3.1. Data and Dependencies

Assemblies for each of the wheat 7A, B and D chromosomes, including the syntenic builds and extra contigs, were generated as described by Berkman *et al.* [18] and used as references for variety specific read mapping. The latest versions of the syntenic builds are accessible at the wheatgenome.info web site [20,27]. The assembly for chromosome 4AL was kindly provided by Dr Pilar Hernandez [32]. Whole genome Illumina sequence data was downloaded from the bioplatforms web site [21,38]. 

The SGSautoSNP pipeline is implemented in Python 2.7 and runs from the command line on any operating system where Python is available. Most of the SGSautoSNP scripts are multithreaded to improve performance with large genomes. Other requirements are pysam [39], a Python module for SAM/BAM formats; Biopython [40,41]; SAMtools version [42] and soap2sam.pl [43] to covert SOAP results to SAM format.

### 3.2. Read Mapping

All Illumina paired-end reads from each cultivar were aligned to the combined assemblies representing 4AL, 7A, 7B and 7D references using SOAPaligner 2 [33] with the –r 0 option and soap_wrapper.py. Depending on data volumes and compute infrastructure, read mapping generally takes between 3 and 48 hours. SOAP generates three results files for each cultivar: paired-end; single mapped reads; and unmapped reads. Only mapped paired reads were used for further analysis. Each of paired mapped read files was converted to sorted and indexed BAM files using SOAP2BAM.py. In order to allow SGSautoSNP.py to detect different cultivars, each read ID in the BAM file was modified to include a cultivar reference tag using generate_subset_BAM.py. Finally, BAM files for each cultivar were merged using SAMtools [42] to produce three BAM files representing 7A, 7B and 7D.

### 3.3. SNP Discovery

The reference is used to assemble the reads, and SNPs are then called between these assembled reads. Depending on data volumes and compute infrastructure, SNP discovery generally takes between 1 and 10 hours. The SGSautoSNP algorithm uses two steps to call a SNP at each locus. Primary SNP calling requires a SNP redundancy score of at least 2. The SNP redundancy score is the minimum number of reads calling the SNP allele at the locus. As at least 2 reads are required for at least 2 cultivars to call a SNP, the minimum coverage at a locus to call a SNP is 4. After this initial SNP call, the algorithm asks if all bases within each cultivar at a locus are the same, which would be expected for homozygous genomes. This process identifies erroneously called SNPs that are due to mis-mapping of reads. 

SGSautoSNP.py produces five output types. A statistics file with the file extension ‘.stat’ contains SNP calling statistics including: (i) scaffold name (ii) SNP number (iii) SNP types (transitions and transversions) (iv) scaffold length. The end of this file contains a summary of all scaffolds. The first results file with the extension ‘.snp’ contains human readable SNP information in text format which can be easily parsed to other formats. Information includes: (i) scaffold name (ii) SNP position on the scaffold (iii) SNP position on the chromosome (iv) SNP score (v) genotypes (which base and how many appear in a particular cultivar) (vi) allele. Three further results formats are produced. VCF [44] files are created to allow the user to view the SNPs in MagicViewer [45]. GFF3 format results are produced for viewing in the GBrowse generic genome browser [46] and Tablet [47], while Flapjack format files (.map and .extension) enable visualisation in Flapjack [48]. 

### 3.4. SNP Filtering

While SNP calling may use many individuals, SNPs that differentiate between two specific individuals are frequently required for downstream analysis. The filter_SNPs.py script parses the text ‘.snp’ file to retrieve SNPs between specific individuals of interest. This script generates the same format output files as SGSautoSNP.py, but specifically for SNPs between two defined individuals. This script also produces a .matrix file which details the number of SNPs between all combinations of cultivars.

### 3.5. Generating a Consensus Sequence

The Bam2ConsensusSequence.py script accepts the alignment in BAM format and generates a consensus sequence for each scaffold. Using multiple CPU cores, the script goes through all nucleotide positions and generates a consensus sequence using the following rules: (i) if no base exists at the position then a N will be inserted; (ii) if only a single read covers the locus then the algorithm uses this read sequence (iii) if more than one read covers the position, and all nucleotides are the same, this nucleotide will be inserted; (iv) if more than one read covers the position, and one single read conflicts with the others, this single read is assumed to be an error and ignored, the majority base is inserted; (iv) if more than one read covers the position, and more than one read conflicts with the others, a degenerate base is inserted using IUPAC notation. The output file is one multiple FASTA file which include all contigs in the original BAM file.

### 3.6. Generating Illumina Marker Assay Files

The SGSautoSNP method can generate Illumina marker assay files for the design of Illumina Infinium and GoldenGate genotyping arrays. The SNP2Markers.py script requires as an input file the consensus sequence in FASTA format generated by Bam2ConsensusSequence.py and the text SNP file with a ‘.snp’ extension generated by SGSautoSNP.py. Additional parameters include (i) species (ii) number of cultivars (iii) SNP library name (iv) version number (v) chromosome name (vi) output directory for the results files.

The script extracts the 5' and 3' sequence surrounding each predicted SNP in the following way: (i) the nucleotide sequence 150 bases each side of the SNP is extracted together with the SNP position in the format [A/C]; (ii) as the Illumina GoldenGate and Infinium assays design probes up to 60 bp adjacent to the SNP, assays are discarded if this region contains any N characters within the consensus sequence. Output files include a file of summary statistics ‘*_marker.stat’ and a marker assay file for input into the Illumina SNP assay design ‘*_GoldenDB.csv’.

### 3.7. Validation

A total of 40 SNPs were randomly selected from the three group 7 reference genomes for validation, representing 18, 9 and 13 SNPs from the A, B and D genomes respectively. These SNPs had a range of redundancy scores. Genomic DNA was isolated from the four wheat cultivars Drysdale, Gladius, Excalibur and RAC875, according to a protocol adapted from [49]. PCR amplification of the 40 loci was performed using primers designed to conserved sequence surrounding the SNPs (Table 2) in a 20 μL reaction volume containing 1 × iTaq PCR buffer (containing 100 mM Tris-HCl and 500 mM KCl, pH 8.3) (Bio-Rad), 200 μM each dNTP (Bio-Rad), 0.5 μM each primer, 1.5 U iTaq DNA polymerase (Bio-Rad), RNase and DNase free water (Gibco) and 60 ng DNA. Thermocycling conditions for the reaction were 94 °C for 2 min, followed by 35 cycles of: 94 °C for 30 s, annealing for 1 min at 60 °C and extension for 1 min at 72 °C. Final extension was performed at 72 °C for 10 min. Gel electrophoresis on a 1% (w/v) agarose gel in 1 × TAE buffer [50] containing ethidium bromide resolved products, which were excised and purified using a silica method based on [51]. 

The purified PCR products were Sanger sequenced using BigDye 3.1, using forward PCR primers, and analysed using an ABI3730xl. The sequences for each locus and cultivar were aligned and compared using Geneious Pro v5.4.6 [52] with a cost matrix of 65%, a gap open penalty of 6, and a gap extension penalty of 3, and each of the SNPs assessed.

## 4. Conclusions

We have developed a pipeline for the identification of single nucleotide polymorphisms in complex genomes using second generation DNA sequence data. The application of this pipeline to wheat identified more than 800,000 SNPs across the three homoeologous group 7 chromosomes 7A, 7B and 7D with a validation rate of greater than 93%. This provides an unprecedented resource for wheat genomics and diversity analysis and demonstrates the potential to expand this application for SNP discovery in other large and complex genomes. SGSautoSNP is freely available on request for academic use.
